# Obinutuzumab, an Anti-CD20, in Refractory Adult Autoimmune Podocytopathies: Report of 2 Cases

**DOI:** 10.1016/j.xkme.2025.101057

**Published:** 2025-06-24

**Authors:** Thomas Loeffler, Adrien Flahault, Asma Alla, Luc Frimat, Raphaël Kormann

**Affiliations:** 1 Department of Nephrology, Centre Hospitalier Régional Universitaire de Nancy, Vandoeuvre-lès-Nancy, France; 2 Université de Lorraine, Vandœuvre-lès-Nancy, France; 3 UMR 1319 INSPIIRE, INSERM, Vandoeuvre-lès-Nancy, France; 4 Nephrolor Network of Care, Vandœuvre-lès-Nancy, France

**Keywords:** Nephrotic syndrome, autoimmune podocytopathies, steroid dependent nephrotic syndrome, steroid resistant nephrotic syndrome, rituximab, obinutuzumab, case report

## Abstract

Autoimmune podocytopathies can represent a therapeutic challenge in the case of frequently relapsing nephrotic syndrome leading to steroid dependence or steroid resistance. Rituximab is an effective treatment option, but allergic reactions or resistance to treatment are frequent. The first case describes a 32-year-old man with corticosteroid-dependent nephrotic syndrome since childhood. After multiple relapses and treatments with cyclosporine, mycophenolate mofetil, and rituximab, a third rituximab treatment led to life-threatening laryngospasm, contraindicating its further use. A treatment with obinutuzumab sustained remission, without additional immunosuppressive treatment, still maintained after 36 months of follow-up. The second case involves a 42-year-old man with nephrotic syndrome, kidney failure, and severe hypertension. A first biopsy showed minimal change disease, whereas a second biopsy found focal segmental glomerulosclerosis lesions. Despite treatments with corticosteroids, cyclosporine, and rituximab, he required hemodialysis. A few years later, he received his first kidney transplant. A relapse was diagnosed at day 3 posttransplantation. Obinutuzumab treatment led to significant improvement in kidney function and sustained remission of the nephrotic syndrome, without relapse 2 years after this treatment. Obinutuzumab could therefore provide a safe and effective alternative to these patients with allergic reactions or resistance to rituximab.

Autoimmune podocytopathies are a newly described entity that encompasses idiopathic nephrotic syndromes (NS), often corticosteroid-dependent or corticosteroid-resistant, and which can recur after kidney transplantation.^1^ Their histologic manifestations include minimal change disease (MCD) and focal segmental glomerulosclerosis (FSGS). They represent a therapeutic challenge in the case of frequently relapsing (FRNS) leading to steroid dependence or steroid resistance.

Clinical evidence of the efficacy of B-cell depletion through rituximab, a monoclonal anti-CD20 antibody, in idiopathic NS, highlights the pathogenic role of B-cells in the disease.^2^ Recent studies suggest the pathogenicity of autoantibodies targeted against podocyte antigens, such as antinephrin antibodies.^3^ A murine model provides proof of concept in favor of antinephrin pathogenicity.^4^ The antinephrin level correlates with disease activity,^4^^,^^5^ such as relapse after kidney transplantation and the detection of these antibodies in the serum is associated in the kidney biopsy with podocyte-associated punctate IgG and disruption of the podocyte cytoskeleton.^4^^,^^5^ Antinephrin serum levels decrease after rituximab treatment and are correlated with the clinical response to this treatment.^4^

Patients developing resistance or antirituximab antibodies after repeated treatments or severe allergic reactions have spurred the search for new molecules targeting CD20.^6^ Moreover, some patients may present with frequent relapse and shorter time of complete remission after repeated rituximab treatment, thus requiring alternative agent.^7^ Obinutuzumab is a type II humanized anti-CD20 monoclonal antibody that induces direct cell death through antibody-dependent cellular cytotoxicity.^7^ Because of a different mode of action and pharmacodynamics, obinutuzumab seems to provide superior therapeutic responses to rituximab.^8^, ^9^, ^10^ This is probably partly because of a deeper and more enduring B-cell depletion with obinutuzumab than with rituximab,^8^ while reducing the prevalence of allergic events and resistance to this treatment.^6^ In a retrospective study, obinutuzumab, used because of rituximab resistance or relapse after rituximab, was associated with encouraging results in FRNS and corticosteroid-dependent idiopathic NS in children.^11^ Data are scarce, however, regarding the use of obinutuzumab in adult patients with FRNS or steroid dependence or steroid resistance. We here report the use of obinutuzumab in one patient with FRNS and one with NS recurrence after transplantation.

## Cases Presentation

### Case 1

The patient was diagnosed with idiopathic NS at age of 7 years. A kidney biopsy showed MCD. He experienced numerous relapses, with steroid dependence, leading to the initiation of long-term cyclosporine treatment. At the age of 16 years, the treatment was modified for mycophenolate mofetil (MMF). At age of 18 years, after another relapse, a first rituximab treatment led to complete remission for 3 years. He received a second rituximab treatment at age of 21 years as he had a new relapse. A third rituximab treatment at age of 23 years led to life-threatening laryngospasm, contraindicating its further use. After this serious adverse event, the MMF treatment was resumed, but continued for less than a year because of significant diarrhea, and the patient was then lost to follow-up.

At age of 26 years, he presented a severe NS relapse with extensive portal venous thrombosis, and a kidney biopsy ruled out FSGS. Management included high doses of corticosteroid and MMF. At age of 27 years, a new relapse occurred with anasarca, predominantly in the form of abundant ascites. Portal hypertension was evident, such as grade III esophageal varices, secondary to the previous portal vein thrombosis complicated by portal cavernoma. Intravenous cyclosporine treatment followed by oral relay was initiated. Cyclosporine level was between 200 and 300 μg/L. Very slow and gradual reduction in corticosteroid and cyclosporine doses resulted in slow clinical-biological improvement.

**Figure 1:**
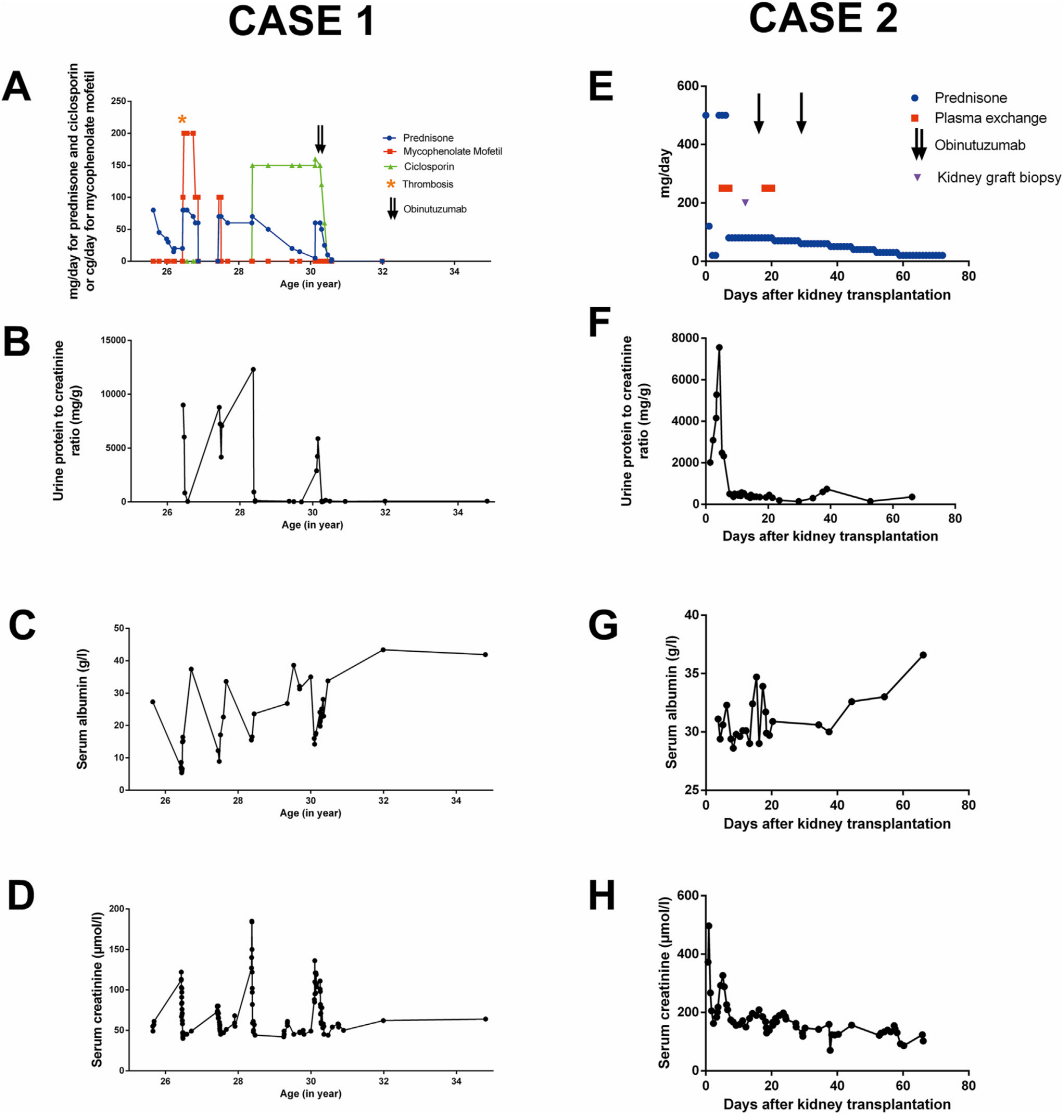
Case 1: evolution from age 25-34 years of doses and type of treatments (A) for the corticosteroid-dependent nephrotic syndrome, urine protein to creatinine ratio (B), serum albumin (C), and serum creatinine (D). The star represents the day of the diagnosis of extensive portal venous thrombosis described in the case. The arrows represent the days of obinutuzumab injections (1,000 mg). Case 2: evolution in days after the kidney transplantation for autoimmune podocytopathy of doses and type of treatments (E), urine protein to creatinine ratio (F), serum albumin (G), and serum creatinine (H). The triangle represents the day of graft kidney biopsy, whereas the arrows represent the days of obinutuzumab injections (1,000 mg).

A new NS flare occurred at age of 30 years in the context of ascitic fluid infection. Corticosteroid therapy at 1 mg/kg was resumed, and cyclosporine treatment was maintained. After the control of proteinuria, obinutuzumab treatment (1,000 mg on days 1 and 15) was initiated to sustain remission, with simultaneous tapering of corticosteroid and cyclosporine treatments (Fig 1). The obinutuzumab treatment was well-tolerated. The following month, the patient developed acute respiratory distress syndrome because of SARS-CoV2, necessitating accelerated tapering of corticosteroid and cyclosporine therapy, with a favorable outcome. The circulating B-cell population was still undetectable 8 months later but normalized 2 years after treatment. Sixteen months after treatment, the immunoglobulin level was low at 6.0 g/L, but within normal range before the second year. Ultimately, 36 months after this last sequence of treatments, the patient is still in complete remission without any further immunosuppressive treatment (Fig 1, case 1).

### Case 2

A 33-year-old man was admitted to the nephrology department for NS. He had proteinuria of 8 g/24 hour, hypoalbuminemia at 19 g/L, normal kidney function, microscopic hematuria, and severe hypertension. A kidney biopsy showed MCD. Corticosteroid therapy at 1 mg/kg resulted in partial remission, with persistent proteinuria at 2.5 g/24 hour and normalized albumin levels. Two months later, he relapsed while on 55 mg/day of corticosteroids, and an acute kidney injury was diagnosed with serum creatinine at 24 mg/L. Cyclosporine at 300 mg/day was added. After 3 months, because of persistent NS and worsening kidney failure, he received a 4-dose rituximab cycle. Despite treatment, his condition worsened, leading to hospitalization for edema and initiation of hemodialysis. A biopsy revealed 24 glomeruli with 4 sclerosed, 5 with FSGS lesions, 40% interstitial fibrosis, and diffuse podocyte foot fusion. He remained treated with hemodialysis for 5 years without additional events.

At age 40 years, he received a living donor kidney transplant with a regimen of tacrolimus, MMF, and long-term corticosteroids. The graft functioned immediately, but on day 3, he had acute kidney injury, serum creatinine at 37 mg/L, and NS recurrence with proteinuria at 7.5 g/g and albuminemia at 30 g/L. A biopsy showed turgescent podocytes, evidence for early recurrence of FSGS. He was treated with solumedrol, prednisone, plasma exchange, and obinutuzumab (1,000 mg on days 1 and 15) (Fig 1). Although proteinuria was already low, the obinutuzumab treatment led to long-lasting recovery, without the need for plasma exchange maintenance therapy (Fig 1, case 2). Two months after kidney transplant, the patient was hospitalized for diarrhea and febrile neutropenia. Four months later, he received amoxicillin for 5 days for pneumonia, and 18 months later he was treated for *Bordetella pertussis* infection. The circulating B-cell population was undetectable at 6 months and within normal range 2 years after treatment. Regarding immunoglobulin, the level was low at 3.7 g/L at 2 months, and within normal range 16 months after treatment. The patient had no relapse, with stable kidney function and serum creatinine at 12 mg/L and no proteinuria more than 2 years after this treatment.

## Discussion

Through these 2 cases, we have highlighted the therapeutic challenge that autoimmune podocytopathies can create, especially when there is resistance or contraindication to rituximab. Specifically, prolonged use of steroids and the use of other immunosuppressive agents such as calcineurin inhibitors is associated with poor tolerance and a high risk of severe infections.

Obinutuzumab was therefore used in both cases according to an induction schedule of 1,000 mg with a 15-day interval.^12^ This treatment was administered when both cases were in early remission from their NS. It was associated with the maintenance of complete remission (negative proteinuria, normalization of albumin levels, and improvement of kidney function) and allowed the rapid discontinuation of other immunosuppressive treatments. Although no additional obinutuzumab treatment was administered, we observed no NS relapse in these 2 patients after 2 to 3 years of follow-up.

Antirituximab antibodies were not measured in either case. In the first case, they were not necessary because we used obinutuzumab rather than rituximab because of the occurrence of an allergic reaction to rituximab. In the second case, because relapse occurred after induction therapy for kidney transplantation, we do not believe that antirituximab antibody testing would have been reliable. Given the disease history, we considered the patient to be resistant to rituximab.

Three other cases of successful treatment with obinutuzumab in autoimmune podocytopathies have already been described. A first case reports the efficacy of obinutuzumab in a corticosteroid-dependent MCD that was resistant to rituximab.^13^ A second case shows the successful rescue of a kidney graft from recurrent FSGS using a combination of obinutuzumab and daratumumab (an anti-CD38 agent).^14^ In the third case, a rituximab-refractory 15-year-old adolescent received a single dose of obinutuzumab, allowing sustained relapse-free remission up to the last follow-up at 18 month.^15^

In this study, we further show that obinutuzumab can be safely and efficiently used in patients with allergic reaction to rituximab contraindicating its use and in the posttransplantation recurrence of a rituximab-resistant NS.

These observations suggest that obinutuzumab could be evaluated in larger-scale studies in the treatment of adult glomerulopathies, especially in autoimmune podocytopathies like MCD and FSGS. Indeed, a single-center, phase 2 open-label trial evaluating the efficacy and safety of obinutuzumab in the treatment of primary FSGS is currently underway (NCT04983888).

These diseases often affect young adults with frequently adverse outcomes, lack of effective and well-tolerated treatments, significant corticosteroid use, which results in significant complications and an impact on the quality of life.^16^ Rituximab has proven to be a potentially effective treatment in these pathologies, but its use can be limited by the occurrence of severe allergic reactions or immunogenicity leading to resistance to it.^6^ Obinutuzumab could therefore provide a safe and effective alternative to these patients.
